# Determination of Major Endogenous FAHFAs in Healthy Human Circulation: The Correlations with Several Circulating Cardiovascular-Related Biomarkers and Anti-Inflammatory Effects on RAW 264.7 Cells

**DOI:** 10.3390/biom10121689

**Published:** 2020-12-17

**Authors:** Rachmad Anres Dongoran, Tsung-Jen Lin, Akhsholphan Byekyet, Sheau-Chung Tang, Jen-Hung Yang, Chin-Hung Liu

**Affiliations:** 1 Program in Pharmacology and Toxicology, School of Medicine, Tzu Chi University, Hualien 970004, Taiwan; 104721108@gms.tcu.edu.tw (R.A.D.); 104752101@gms.tcu.edu.tw (T.-J.L.); 107721103@gms.tcu.edu.tw (A.B.); 2 Indonesian Food and Drug Authority (Indonesian FDA), Jakarta 10560, Indonesia; 3 Department of Nursing, National Taichung University of Science and Technology, Taichung 40640, Taiwan; s6160051@gmail.com; 4 Department of Dermatology, Changhua Christian Hospital, Changhua 500, Taiwan; jh1000521@gmail.com; 5 Department of Pharmacology, School of Medicine, Tzu Chi University, Hualien 970004, Taiwan

**Keywords:** FAHFAs, 9-POHSA, 9-OAHSA, cardiovascular disease, anti-inflammatory, SAH, TMAO, l-carnitine

## Abstract

Fatty acid esters of hydroxy fatty acids (FAHFAs) are newly discovered long-chain fatty acids. However, the major endogenous FAHFAs in healthy human circulation, their correlation with cardiovascular (CV) biomarkers, and their anti-inflammatory effects have not been investigated and remain unclear. In the present study, a total of 57 healthy subjects were recruited. Liquid chromatography–mass spectrometry (LC-MS) was developed for the simultaneous determination of seven FAHFAs, four long-chain fatty acids, and four non-traditional circulating CV-related biomarkers. We found two major types of FAHFAs in healthy human circulation, palmitoleic acid ester of 9-hydroxystearic acid (9-POHSA), and oleic acid ester of 9-hydroxystearic acid (9-OAHSA). Both 9-POHSA and 9-OAHSA had a strong positive correlation with each other and were negatively correlated with fasting blood glucose, *S*-adenosyl-l-homocysteine (SAH)*,* and trimethylamine *N*-oxide (TMAO), but not with l-homocysteine. 9-POHSA was also positively correlated with l-carnitine. Moreover, we confirmed that both 9-POHSA and 9-OAHSA exhibited an anti-inflammatory effect by suppressing LPS stimulated cytokines, including *IL-1β* and *IL-6* in RAW 264.7 cells. In addition, palmitoleic acid also had a positive correlation with 9-POHSA and 9-OAHSA. As far as we know, this is the first report showing the major endogenous FAHFAs in healthy subjects and their CV protection potential which might be correlated with SAH and TMAO reduction, l-Carnitine elevation, and their anti-inflammatory effects.

## 1. Introduction

Branched fatty acid esters of hydroxy fatty acids (FAHFAs) are a new class of endogenous bioactive lipids that have been found to be enriched in adipose tissue and circulation [1]. In chemical synthesis, the esterification of FAHFAs could be derived from several fatty acids (FA), including palmitic acid (C16:0, PA), palmitoleic acid (C16:1*n*-7, POA), stearic acid (C18:0, SA), or oleic acid (C18:1*n*-9, OA) with their corresponding hydroxylated fatty acid (HFA) to form a palmitic acid ester of 5-hydroxystearic acid (5-PAHSA), palmitic acid ester of 9-hydroxystearic acid (9-PAHSA), palmitic acid ester of 12-hydroxystearic acid (12-PAHSA), palmitic acid ester of 9-hydroxypalmitic acid (9-PAHPA), stearic acid ester of 9-hydroxystearic acid (9-SAHSA), palmitoleic acid ester of 9-hydroxystearic acid (9-POHSA), and oleic acid ester of 9-hydroxystearicacid (9-OAHSA), etc. [2], and the different bonding position of single carbon branch defines different structural regioisomers (e.g., 5-PAHSA, 9-PAHSA or 12-PAHSA). FAHFA-specific hydrolases, androgen-induced gene 1 protein (AIG1), androgen-dependent TFPI-regulating protein (ADTRP), and carboxyl ester lipase (CEL) have been identified in vivo [3,4]; however, the endogenous biosynthesis pathway of FAHFAs has not been well investigated [1,2]. The synthesis occurs de novo in tissues catalyzed by a fatty acyltransferase to transfer a fatty acid to an HFA [1]. Kuda et al. [5] reported that the levels of DHAHLA, LAHDHA, DHAHDHA, and some other FAHFAs derived from omega-3 fatty acids supplementation, i.e., linoleic acid (LA) and docosahexaenoic acid (DHA) increased after omega-3 fatty acids administration in diabetic patients and obese mice. To date, including all the regioisomers, hundreds of known FAHFAs have been identified [6], but only a few of these have been investigated for their biological activity [1,5,7,8,9,10]. Therefore, there is a need to explore the importance of the biological activities of various FAHFAs in the human body.

Endogenous FAHFAs have also exhibited type 2 diabetes treatment potential and anti-inflammatory effects in several previous studies [1,5,8]. PAHSAs, which are among the most studied, have been reported to reduce the blood glucose levels and improve insulin sensitivity in an animal model [1]. In addition, 9-PAHSA also inhibited LPS-induced pro-inflammatory cytokine production in a macrophage-like cell line (RAW 264.7 cells) [5]. Moreover, administration of 9-PAHSA lowered the levels of inflammatory macrophages in the adipose tissue of mice on a high-fat diet [1], and reduced clinical and pathological disease severity in a mouse model of colitis [7].

FAHFAs are newly discovered branched fatty acids; however, the major endogenous FAHFAs in healthy human circulation and their correlation with cardiovascular (CV) biomarkers have not been investigated. CVDs present the highest risk of mortality globally [11,12]. Traditional risk factors, including diabetes mellitus (DM), obesity, hypertension, arteriosclerosis, higher triglyceride (TG), total cholesterol (TCH), low-density lipoprotein cholesterol (LDL-C), and reduction in high-density lipoprotein cholesterol (HDL-C), may lead to the development of CVD [13,14,15]. The fundamental role of inflammation has been proved in basic and clinical investigations in CVD [16,17]. Inflammatory cytokines and vascular imaging support the systemic and diffuse nature of inflammation associated with CVD [18,19]. Several studies have also shown that low-grade systemic inflammation leads to an increased risk of CVD [19,20,21]. The common inflammatory cytokines include interleukin-1β (IL-1β) and interleukin-6 (IL-6) which indicate greater susceptibility to the development of CVD [22,23,24]. Therefore, to investigate the CVDs protection effect of FAHFAs, we explored the correlation of major endogenous FAHFAS with several cardiovascular biomarkers and their anti-inflammatory effects on RAW 264.7 macrophage cells.

In the present study, liquid chromatography–mass spectrometry (LC-MS) was developed for the determination of seven representative types of FAHFAs (9-POHSA, 9-OAHSA, 5-PAHSA, 9-PAHSA, 12-PAHSA, 9-PAHPA, and 9-SAHSA), four long-chain fatty acids (PA, SA, POA, and OA), which are endogenous precursors of FAHFAs, and four non-traditional circulating CV-related biomarkers [l-homocysteine (l-Hcy), *S*-adenosyl-l-homocysteine (SAH), l-carnitine (l-Car), and trimethylamine *N*-oxide (TMAO)] in plasma of a total 57 healthy subjects. The main purpose of this study was to investigate the major FAHFA in healthy subjects, their association with CV-related biomarkers, and further explore their anti-inflammatory effects in RAW 264.7 murine macrophage cell line. Our results showed that the major types of FAHFAs in healthy human circulation were 9-POHSA and 9-OAHSA. Both 9-POHSA and 9-OAHSA had a significant correlation with CV protection and possessed anti-inflammatory effects. In addition, increasing the level of endogenous POA might be correlated with increase the levels of both FAHFAs.

## 2. Materials and Methods 

### 2.1. Participants

**Figure 1:**
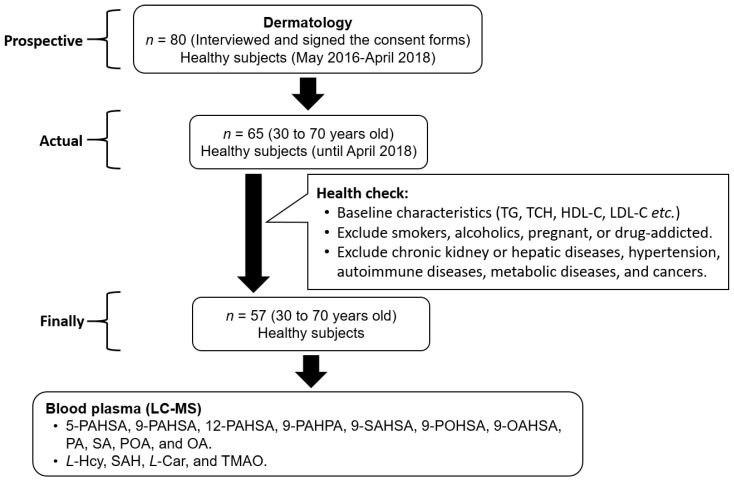
Flow diagram of healthy subject enrollment.

In the beginning, the prospective enrollment number was 80 healthy participants. After baseline characteristics and health criteria selection, a total of 57 healthy subjects were enrolled from the Department of Dermatology, Buddhist Tzu-Chi General Hospital from May 2016 to April 2018. The baseline characteristics, including age, height, body weight, and body mass index (BMI), were measured or calculated. To ensure a healthy status, the subjects were interviewed to know their medical history. We excluded subjects who were smokers, alcoholics, pregnant, or drug-addicted. Additionally, the selection criteria were added including participants without chronic kidney or hepatic diseases, hypertension, autoimmune diseases, metabolic diseases, and cancers. The age of subjects has been restricted from 30 to 70 years old (Figure 1). This study was approved by the ethics committee of Buddhist Tzu-Chi General Hospital Taiwan (IRB105-137-B) and carried out in accordance with the Declaration of Helsinki. All of the subjects have signed the consent forms and agreed to this study and fasting whole blood was then collected from each subject.

### 2.2. Biochemical Analysis

The blood samples (5 mL) were collected by using Vacutainer K2E-EDTA tubes and transferred into 15 mL centrifuge tube contained Ficoll-paque PLUS medium [hall blood/Ficoll-paque PLUS medium = 4/3 (*v/v*)] (GE Healthcare Life Sciences, Pittsburgh, PA, USA), then centrifuged (3000 rpm, 30 min). After centrifugation, the supernatant was collected in sterile tubes and 1 N of acetic acid was added to prevent degradation. All the samples were stored at −80 °C and the storage time was less than one year. The plasma levels of fasting blood glucose (Glu-AC) and lipid profiles (TG, TCH, HDL-C, and LDL-C) were determined using the COBAS Integra 800 autoanalyzer (Roche Diagnostics, Basel, Switzerland) [25,26].

### 2.3. Standards Preparation and Calibration Curves

The standard stock solutions of 5-PAHSA, 9-PAHSA, 12-PAHSA, 9-PAHPA, 9-SAHSA, 9-POHSA, 9-OAHSA, PA, SA, POA, and OA were purchased from Cayman Chemical (Ann Arbor, MI, USA). The standards of l-Hcy, SAH, l-Car, and TMAO (Sigma Aldrich, St. Louis, MO, USA) were dissolved in double-distilled water (ddH_2_O) or HPLC grade methanol (MeOH) with final concentration 0.1 mg/mL for stock solution. The stable isotopes ^13^C_16_-palmitic acid (^13^C_16_-PA) and d3, l-methionine (d3-l-Met) were obtained from Sigma Aldrich (St. Louis, MO, USA), and the d9-TMAO was purchased from Cambridge Isotope Laboratories (Andover, MA, USA). All of the stock solutions and stable isotopes solution were stored at −20 °C and the storage time was less than one year. The calibration curves were prepared by using stock dilution and formulated by using the peak area ratio of the analytical standards and the internal standards.

### 2.4. Extraction and Determination of the FAHFAs and Fatty Acids

After thawing at room temperature (RT) for 10–20 min, 100 µL plasma was transferred into the new 1.5 mL centrifuge tube. Deproteinization was done by the addition of 3 parts of methanol and incubated in RT for 20 min. The supernatant was collected in a glass vial after being centrifuged at 3000× *g* for 10 min at 4 °C. The supernatant was dried by using the nitrogen gas and the sample was further recombined in 100 µL chloroform. The solid-phase extraction (SPE) columns (Strata-X-C 33 µm polymeric strong cation 60 mg/3 mL, Phenomenex, Torrance, CA, USA) were installed and followed by adjunction of 1.0–1.5 mL hexane to the columns for activation (vacuum for 20–30 s). The recombinant samples were loaded into each column and 1.0–1.5 mL ethyl acetate was added for extraction into the new sample vials (vacuum for 30–45 s). Nitrogen gas was used to dry down all of the samples again and reconstituted in 100 µL methanol. The internal standards were added before deproteinization. Finally, the sample was transferred into an LC-MS sample vial for 30 µL injection.

### 2.5. Extraction and Determination of the l-Hcy, SAH, l-Car, and TMAO

The traditional deproteinized extraction method and the Novum^®^ simplified liquid extraction (SLE) column (Phenomenex, Torrance, CA, USA) extraction method were used for l-Hcy, SAH, l-Car, and TMAO extraction. Briefly, the plasma sample was thawed at room temperature for 10–20 min and 100 µL plasma were transferred into another new 1.5 mL centrifuge tube. The plasma samples were deproteinized by the addition of 3 parts of methanol and incubated at room temperature for 20 min or adjunction of 100 µL of 50 mM Sodium phosphate dibasic heptahydrate (Sigma Aldrich, MO, USA) solution for half dilution and mixed briefly (approximately 5 s). After 20 min, the deproteinization sample was centrifuged at 3000× *g* for 10 min at 4 °C, and the half dilution sample was loaded into the Novum^®^ SLE column with a gentle pulse of vacuum (15–20 s) until the sample moved into the filter valve. After 5 min, 1.2–1.5 mL ethyl acetate was added for extraction (vacuum for 30–45 s), and the supernatant of centrifugation was collected, and the nitrogen gas was used to dry down in parallel. The samples were reconstituted in 100–300 µL methanol and filtered. After being mixed thoroughly (5–10 s) the samples were transferred into LC-MS sample vials for 30 µL injection.

### 2.6. LC-MS Conditions

The Waters e2695 high-performance liquid chromatography (HPLC) system connects with a single quadrupole mass spectrometer (ACQUITY QDa^®^, Waters Corp., Milford, MA, USA) was used for analysis. The Phenomenex Luna^®^ C18(2) column (5 µ, 250 × 4.60 mm, 100 Å) and combination with a guard cartridge system (KJ0-4282, Phenomenex) was used for the separation of analytes. For FAHFAs and fatty acids analysis, the temperature of the column was set at 35 °C and the flow rate of the mobile phase was set at 0.6 mL/min. Mobile phase A was composed of (60: 40 acetonitrile: ddH_2_O) and mobile phase B was composed of (90: 10 isopropanol: acetonitrile). Both mobile phases A and B contained 9.2 mM ammonium acetate (Sigma Aldrich, St. Louis, MO, USA). The gradient of the mobile phase was started at 15% B and changed into 30% B in 8 min, then up to 48% B in another 2 min and to 82% B until 44 min. The 99% B was achieved in 2 min and hold for another 2 min. Subsequently, 15% B started at 48.4 min and was held until 60 min. After analysis, the column was washed by isopropanol and stored to avoid drying. The MS-QDa detector settings were as follows, vaporization temperature 400 °C, capillary voltage 0.8 kV, and sample cone 20.0 V. The LC-MS condition for l-Hcy, SAH, l-Car, and TMAO followed the analysis condition according to our previous study [25]. The single ion recording (SIR) mode was used for FAHFAs, fatty acids, l-Hcy, SAH, l-Car, and TMAO. The FAHFAs and fatty acids, including 5-PAHSA 537.5 *m/z*, 9-PAHSA 537.5 *m/z*, 12-PAHSA 537.5 *m/z*, 9-PAHPA 509.4 *m/z*, 9-SAHSA 565.5 *m/z*, 9-POHSA 535.4 *m/z*, 9-OAHSA 563.5 *m/z*, PA 255.1 *m/z*, POA 253.2 *m/z*, OA 281.2 *m/z*, SA 283.2 *m/z*, and ^13^C_16_-PA 271.4 *m/z* were detected in negative ion mode detection. The l-Hcy 136.1 *m/z*, SAH 385.0 *m/z*, l-Car 162.1 *m/z*, TMAO 76.0 *m/z*, d9-TMAO 85.1 *m/z*, and d3- l-Met 154.1 *m/z* were detected in positive ion mode detection. The LC-MS data were analyzed using LC-MS Empower 3 software (Waters Corp., Milford, MA, USA). The detection results were quantified by peak areas and compared with calibration curves obtained from the standards solution.

### 2.7. Cell Culture

RAW 264.7, mouse macrophage cells, were originally obtained from the American Type Culture Collection (ATTC, Manassas, VA, USA). Cells were cultured in Dulbecco’s modified Eagle medium (DMEM, Gibco, New York, NY, USA) supplemented with 10% fetal bovine serum (FBS, Gibco, USA) and 1% penicillin-streptomycin (Mediatech, Manassas, VA, USA) at 37 °C in a humidified 5% CO_2_ atmosphere.

### 2.8. RNA Isolation and Quantitative Reverse Transcription–Polymerase Chain Reaction (RT-qPCR) Analysis

The mRNA expression was determined by quantitative reverse transcription–polymerase chain reaction (RT-qPCR). RAW 264.7 cells were seeded at a density of 3 × 10^6^ cells into a 10 cm petri dish. The cells were then treated with the indicated concentration of lipopolysaccharide (LPS, 100 ng/mL), 9-PAHSA, 9-POHSA, and 9-OAHSA (2 and 10 µM) or other treatments, where 0.2% dimethyl sulfoxide (DMSO, Sigma Aldrich, St. Louis, MO, USA) was used as a vehicle control group. After 24 h of treatment, total RNA was extracted using the Total RNA Isolation Kit (GeneDirex, Taoyuan, Taiwan) according to the manufacturer’s protocol. The purity and quantity of RNA in each sample was determined using a NanoDrop 2000C Spectrophotometer (Thermo Fisher, Waltham, MA, USA). The GScript First-Strand Synthesis Kit (GeneDirex, Taoyuan, Taiwan) was used to perform reverse transcription from RNA to cDNA according to the manufacturer’s protocol. The primers and annealing temperature of genes analyzed by RT-qPCR are glyceraldehyde 3-phosphate dehydrogenase (*GAPDH*) (F: AGGTCGGTGTGAACGGATTTG, R: TGTAGACCATGTAGTTGAGGTCA, 60 °C), *IL-1β* (F: ACCTGGGCTGTCCTGATGAGAG, R: CCACGGGAAAGACACAGGTAGC, 60 °C), and *IL-6* (F: AACCACCGCCTTCCCTACTT, R: GCCATTGCACAACTCTTTTCTC, 60 °C). *GAPDH* was used as an internal control. The final concentration of cDNA and primers was 100 ng and 900 nM, respectively. Power SYBR Green PCR Master Mix (Thermo Fisher, USA) was used for performing real-time PCR assay. The PCR reaction with 40 cycles (10 min hold at 95 °C, 15 s denature at 95 °C, annealing, and extension 1 m at 60 °C) of amplification was performed using instrument QuantStudio^®^ 5 System (Thermo Fisher, USA) and analyzed using comparative CT (△△CT). Each RT-qPCR was repeated with at least four to five different RNA and cDNA preparations for both control and treated cells [27].

### 2.9. Statistical Analysis

**Figure 2:**
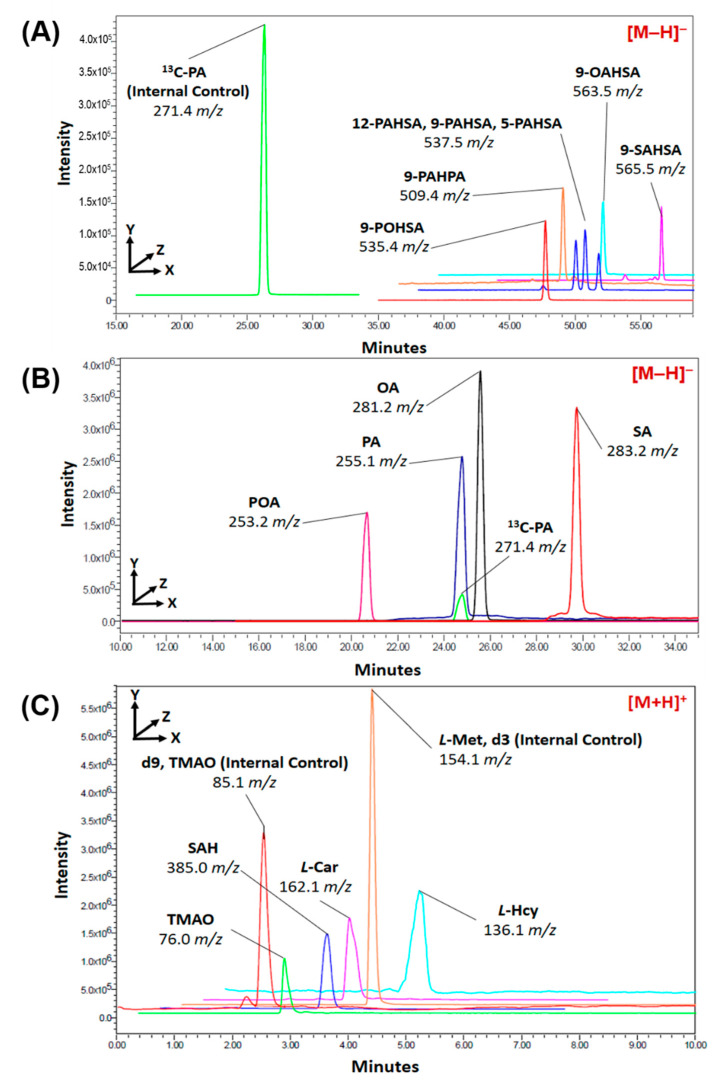
Determination of fatty acid esters of hydroxy fatty acids (FAHFAs), long-chain fatty acids, and cardiovascular (CV)-related biomarkers by using liquid chromatography-mass spectrometry (LC-MS). Seven types of circulating FAHFAs (5-PAHSA, 9-PAHSA, 12-PAHSA, 9-PAHPA, 9-SAHSA, 9-POHSA, and 9-OAHSA) (**A**), four long-chain fatty acids (PA, SA, POA, and OA) (**B**), and four non-traditional circulating CV-related biomarkers (l-Hcy, SAH, l-Car, and TMAO) (**C**) were analyzed by LC-MS.

**Figure 3:**
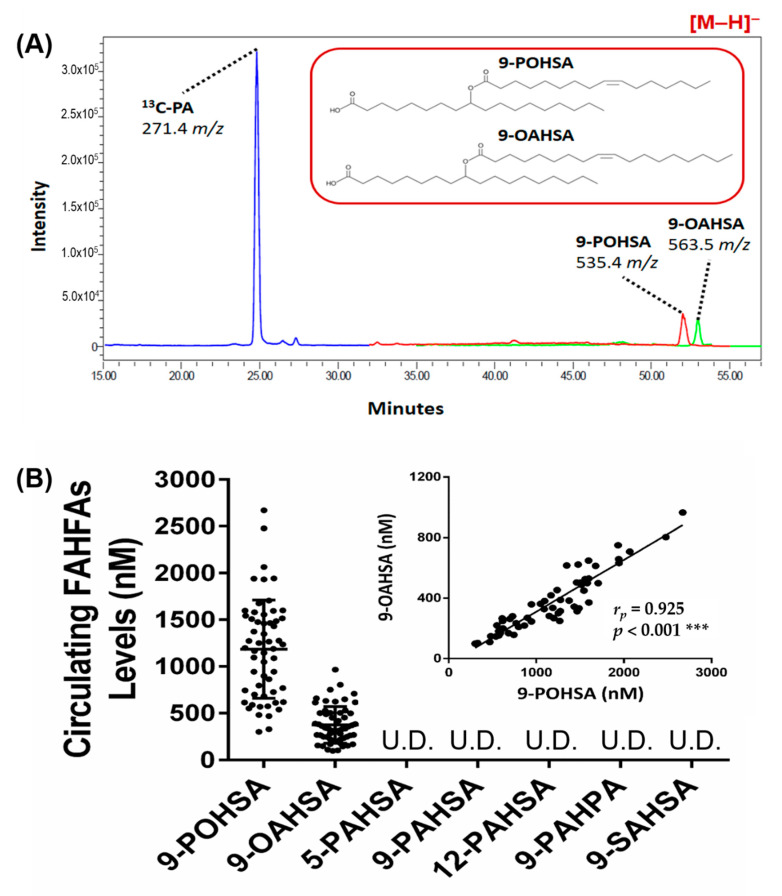
9-POHSA and 9-OAHSA were major endogenous FAHFAs in healthy subjects. Both 9-POHSA and 9-OAHSA were clearly measured in healthy subjects, where 10 μM ^13^C-palmitic acid (PA) was used as an internal standard (**A**). 9-POHSA and 9-OAHSA were major endogenous FAHFAs and had a strong positive correlation with each other (*r* = 0.9254, *p* < 0.001) while the other five types of FAHFAs (5-PAHSA, 9-PAHSA, 12-PAHSA, 9-PAHPA, and 9-SAHSA) were below the limit of detection or undetected (**B**). *r_p_*: Pearson correlation coefficient. Data are presented as mean ± SD, U.D. = undetected (*** *p* < 0.001).

**Figure 4:**
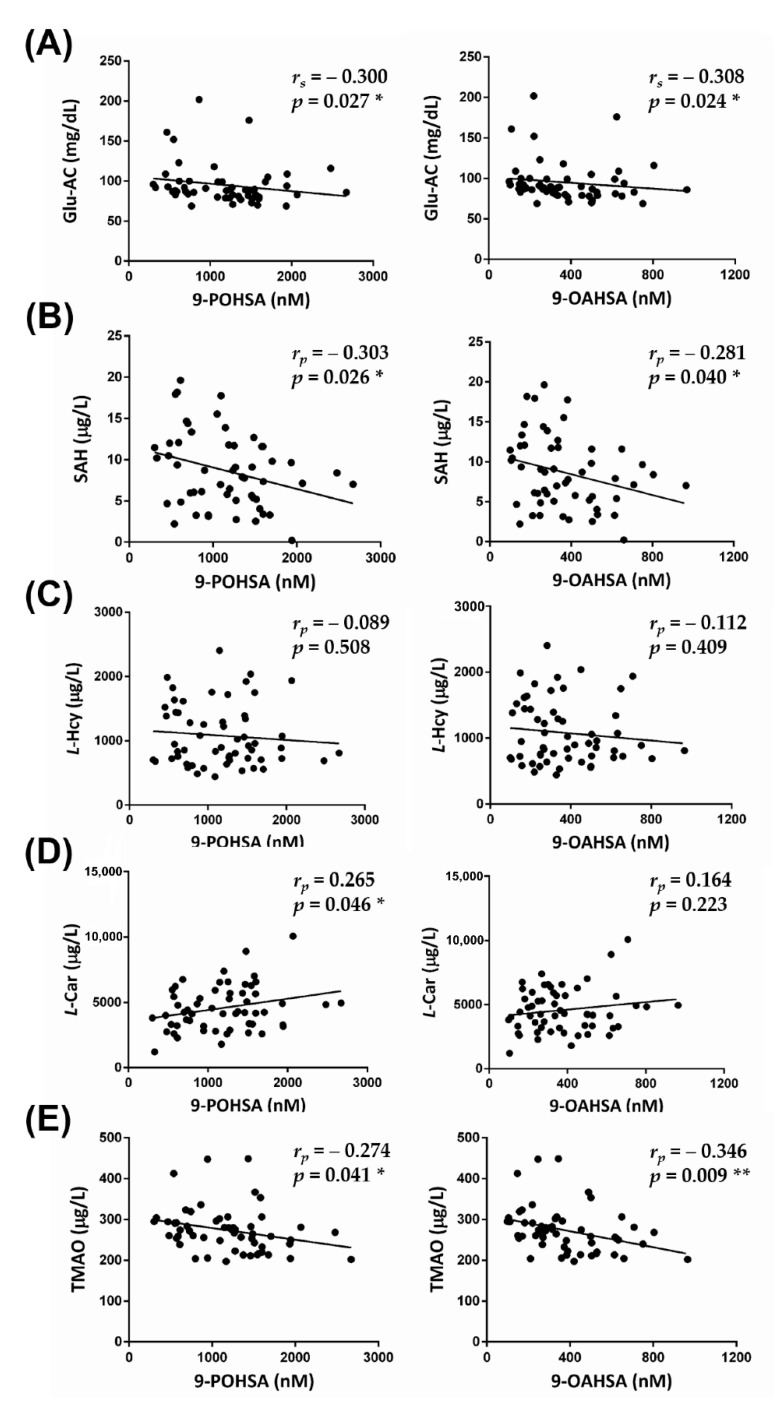
The correlation of FAHFAs with non-traditional CV-related biomarkers. 9-POHSA and 9-OAHSA had a significant negative correlation with Glu-AC (**A**) and SAH (**B**), but not with l-Hcy (**C**). l-Car was also positively correlated with 9-POHSA (**D**). TMAO had a significant negative correlation with both 9-POHSA and 9-OAHSA (**E**). *r_s_*: Spearman correlation coefficient, *r_p_*: Pearson correlation coefficient. Data are presented as mean ± SD (* *p* < 0.05, ** *p* < 0.01).

**Figure 5:**
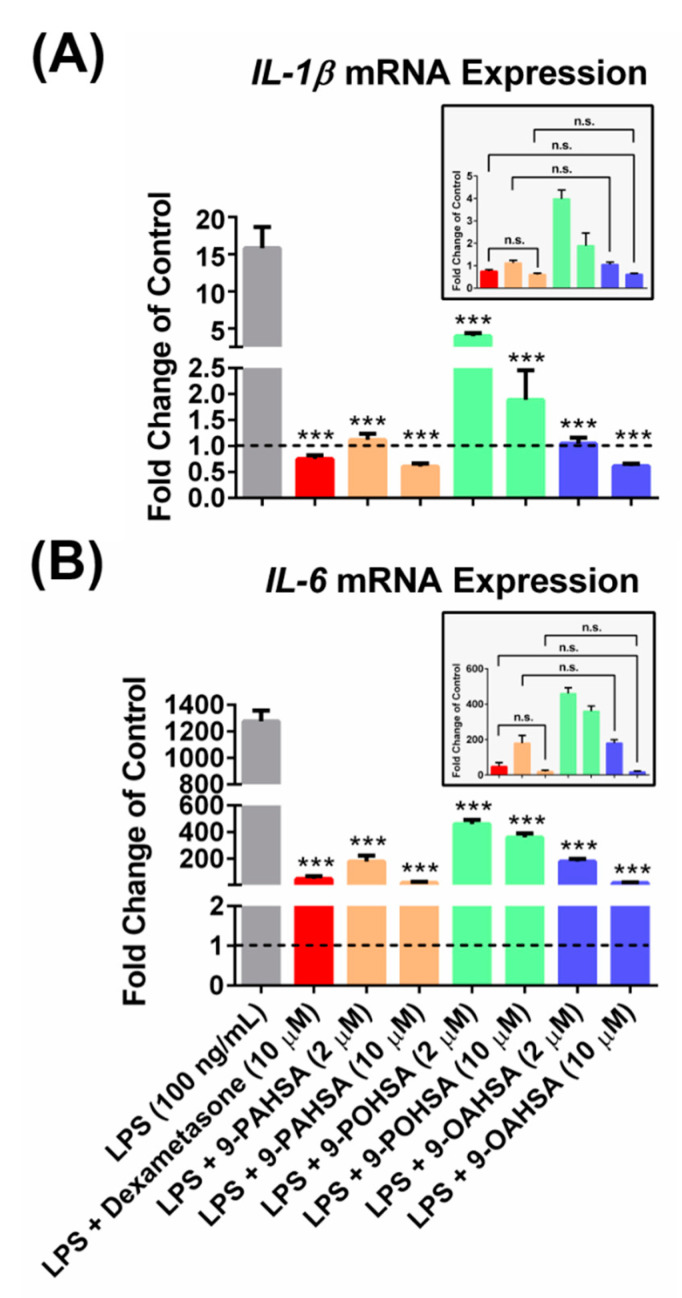
9-POHSA and 9-OAHSA possessed anti-inflammatory effects. RAW 264.7 cells were treated with the indicated concentration of lipopolysaccharide (LPS, 100 ng/mL), 9-PAHSA, 9-POHSA, 9-OAHSA (2 and 10 µM), or co-treatments, where dimethyl sulfoxide (DMSO, Sigma, USA) was used as a vehicle control group. After 24 h, the cells were harvested and further processed for RT-qPCR. 9-POHSA and 9-OAHSA suppressed LPS stimulated cytokines *IL-1β* (**A**) and *IL-6* (**B**) gene expression. The dashed line across all bars to axis X indicated control(s) which assigned as one. Data are presented as mean ± SD, *n* = 4~5, (*** *p* < 0.001, n.s. = not significant).

**Figure 6:**
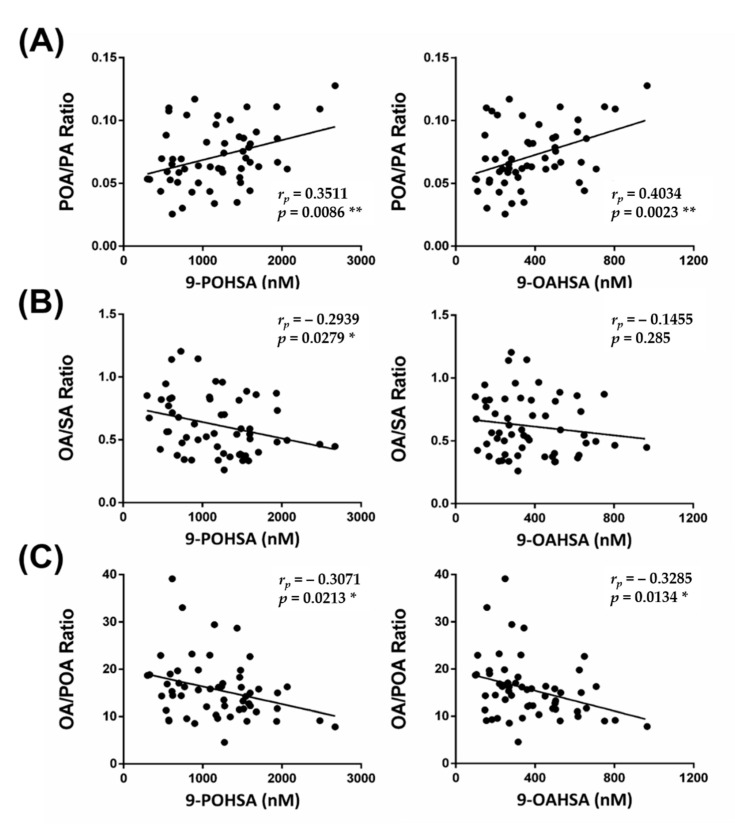
The correlation of FAHFAs with their fatty acid precursor ratio. The correlations between 9-POHSA and 9-OAHSA with POA/PA ratio, OA/SA ratio, and OA/POA ratio are shown in (**A**–**C**), respectively. *r_p_*: Pearson correlation coefficient. Data are presented as mean ± SD (* *p* < 0.05, ** *p* < 0.01).

GraphPad Prism 6.0 (GraphPad Software, San Diego, CA, USA) was used to present the data, perform statistical methods of correlation analysis, and two-tailed Student’s independent *t*-test, as shown in Figure 2, Figure 3, Figure 4, Figure 5 and Figure 6. SPSS for Windows (version 20.0; SPSS Inc., Chicago, IL, USA) was used to check data distribution by using the Shapiro-Wilk normality test. The Pearson correlation coefficient (*r*_p_) and Spearman correlation coefficient (*r*_s_) were used for the normal distribution and non-normal distribution data, respectively. The statistical significance was set at * *p* < 0.05.

## 3. Results

### 3.1. Enrollment and Characteristics of Healthy Subjects

Subjects whose health status had been ensured were considered to be eligible for the study. The baseline characteristics were measured or calculated to confirm the health status of the subjects, as shown in Table 1. A total of 57 subjects were healthy.

### 3.2. Linearity and Plasma Analysis

The gradient system of LC-MS methods was modified from the previous studies [28,29] and renewed according to our ideas. The calibration curves were calculated by using the peak area ratio of the analytical standards and the internal standards (^13^C_16_-PA, d3- l-Met, or d9-TMAO). All of the coefficients of determinations (R^2^) of linearity were greater than 0.995. Seven types of circulating FAHFAs (5-PAHSA, 9-PAHSA, 12-PAHSA, 9-PAHPA, 9-SAHSA, 9-POHSA, and 9-OAHSA), four long-chain fatty acids (PA, SA, POA, and OA) that are endogenous precursors of FAHFAs, and four non-traditional circulating CV-related biomarkers (l-Hcy, SAH, l-Car, and TMAO) were analyzed by LC-MS. The chromatograms are shown in Figure 2, and their levels in healthy subjects are shown in Table 2.

### 3.3. 9-POHSA and 9-OAHSA Were Major Endogenous FAHFAs in Healthy Subjects

We found that both 9-POHSA and 9-OAHSA were major endogenous FAHFAs in healthy subjects, and they were clearly quantified in human circulation (Figure 3A,B). In addition, 9-POHSA (1184.4 ± 526.1 nM) had a higher concentration in plasma than 9-OAHSA (374.0 ± 194.6 nM) (Table 2). The other five types of FAHFAs (5-PAHSA, 9-PAHSA, 12-PAHSA, 9-PAHPA, and 9-SAHSA) were below the limit of detection or were undetected. Moreover, we found that 9-POHSA and 9-OAHSA had a strong positive correlation (*r* = 0.9254, *p* < 0.001) with each other (Figure 3B insert).

### 3.4. The Correlation of FAHFAs with Fasting Blood Glucose and Lipid Profiles

As shown in Figure 4A, both 9-POHSA and 9-OAHSA had a significant negative correlation with Glu-AC (*r* = −0.300, *p* = 0.027 and *r* = −0.308, *p* = 0.024, respectively), but did not have any significant correlation with lipid profiles (TG, TCH, HDL-C, or LDL-C) (data not shown).

### 3.5. The Correlation of FAHFAs with Non-Traditional CV-Related Biomarkers

To investigate the CVD protection of FAHFAs in healthy subjects, we analyzed the correlation of circulating 9-POHSA and 9-OAHSA with four non-traditional circulating CV-related biomarkers. As shown in Figure 4B,C, 9-POHSA (*r* = −0.303, *p* = 0.026) and 9-OAHSA (*r* = −0.281, *p* = 0.040) had a significant negative correlation with SAH, but not with l-Hcy (*r* = −0.089, *p* = 0.508 and *r* = −0.112, *p* = 0.409, respectively). l-Car was also positively correlated with 9-POHSA (*r* = 0.265, *p* = 0.046), but not with 9-OAHSA (*r* = 0.164, *p* = 0.223) (Figure 4D). In addition, TMAO had a significant negative correlation with both 9-POHSA (*r* = −0.274, *p* = 0.041) and 9-OAHSA (*r* = −0.346, *p* = 0.009) (Figure 4E). Together, 9-POHSA and 9-OAHSA had CVD protection in healthy subjects.

### 3.6. 9-POHSA and 9-OAHSA Possessed Anti-Inflammatory Effects on RAW 264.7 Cells

We investigated the anti-inflammatory effects of 9-POHSA and 9-OAHSA on suppression of LPS stimulated cytokines, including IL-1β and IL-6 gene expression in RAW 264.7 cells. To evaluate the anti-inflammatory effects of FAHFAs, we used dexamethasone as a positive control for cytokines inhibition. We also treated the cells with 9-PAHSA since its anti-inflammatory activity has been reported in vitro and in vivo [1,5,7]. As shown in Figure 5, LPS stimulation increased the gene expression of cytokines IL-1β and IL-6, and our data further clearly showed that 2 to 10 µM of 9-PAHSA, 9-POHSA, and 9-OAHSA functioned as 10 µM dexamethasone to suppress LPS stimulated IL-1β and IL-6 gene expression. Compared to dexamethasone, both 9-PAHSA and 9-OAHSA had the same anti-inflammatory potency on IL-1β and IL-6 inhibition. 

### 3.7. The Correlation of FAHFAs with Their Fatty Acid Precursors

The correlation between PA, SA, POA, and OA with 9-POHSA and 9-OAHSA were shown in Figure S1 and Figure 6. Higher POA level (Figure S1C), or when more POA was formed (represented from POA to PA ratio, Figure 6A), had a positive correlation with 9-POHSA (*r* = 0.3511, *p* = 0.0086) and 9-OAHSA (*r* = 0.4034, *p* = 0.0023). However, higher-level OA (Figure S1D), or when more OA was formed (represented from OA to SA and OA to POA ratio, Figure 6B,C), did not contribute to the formation of 9-POHSA (*r* = −0.3071, *p* = 0.0213) and 9-OAHSA (*r* = −0.3285, *p* = 0.0134).

## 4. Discussion

In this study, we found that 9-POHSA and 9-OAHSA were two major types of FAHFAs in healthy human circulation. To investigate the CVD protection of FAHFAs, we analyzed their correlations with CV-related biomarkers. We found that both 9-POHSA and 9-OAHSA had a negative correlation with Glu-AC, SAH, and TMAO, but had no correlation with l-Hcy and lipid profiles (TG, TCH, HDL-C, and LDL-C). 9-POHSA had a positive correlation with l-Car, but 9-OAHSA did not. Increasing the level of endogenous POA in the body might be related to the increases of both FAHFAs. Moreover, 9-POHSA and 9-OAHSA possessed an anti-inflammatory effect on suppression of LPS stimulated cytokines. These results suggested that 9-POHSA and 9-OAHSA may play a role in CV protection. As far as we know, this is the first report showing the major endogenous FAHFAs in healthy humans and their cardiovascular protection effects.

l-Hcy is a well-known risk factor of CVD and has been investigated in previous studies [30,31,32]. However, several studies also indicated that SAH may have a higher potential effect than l-Hcy in atherosclerosis and in early clinical detection of CVD [33,34,35,36]. Therefore, we determined the levels of both l-Hcy and SAH and further investigated their correlations with 9-POHSA and 9-OAHSA. We found that the SAH but not l-Hcy had a significant negative correlation with 9-POHSA and 9-OAHSA. The 9-POHSA and 9-OAHSA might be directly affecting SAH production to further prevent the CVD occurs in healthy humans. It also implies that SAH could be a more sensitive biomarker than l-Hcy in early CVD monitoring.

l-Car is a conditional nutrient involves in energy production, carbohydrates, and long-chain fatty acid transport [37]. Several previous studies exposed the potential of l-Car as antioxidants [38] and promoted HDL-C upregulation on coronary artery disease patients [39,40]. Previous studies indicated that the anti-inflammatory effects of l-Car were achieved through suppression of reactive oxygen species and further inhibition of the nuclear factor kappa-light-chain-enhancer of activated B cells (NF-κB) signaling pathway [41,42]. We found that l-Car had a positive correlation with 9-POHSA but not with 9-OAHSA levels. l-Car might be contributed to upregulate the circulation level of 9-POHSA which could be acquired lipids metabolism and long-chain fatty acids transportation [37,43].

Recently, several studies have found that TMAO is a potential CVD risk factor, and a higher level of l-Car may have caused an increased TMAO concentration into circulation through gut microbiota transformation [44,45,46,47]. Hence, we determined the correlation of TMAO levels with 9-POHSA and 9-OAHSA levels. Our results indicated that TMAO had a negative correlation with both 9-POHSA (*r* = −0.274, *p* = 0.041) and 9-OAHSA (*r* = −0.346, *p* = 0.009). These results suggested that 9-POHSA and 9-OAHSA might be involved in reducing TMAO production and further decreased the accumulation of TMAO in human circulation.

We confirmed that both 9-POHSA and 9-OAHSA exhibited an anti-inflammatory effect by suppressing LPS stimulated cytokines, including *IL-1β* and *IL-6* in RAW 264.7 cells. Similar to our results, several studies have also reported the anti-inflammatory of other FAHFAs in vivo and in vitro [1,5,7,9], especially 9-PAHSA, which is the most investigated among FAHFAs for its anti-inflammatory activity [1,7]. The 9-PAHSA inhibited LPS-induced pro-inflammatory cytokines production in the macrophage-like cell line, lowered the levels of inflammatory macrophages in the adipose tissue of mice on a high-fat diet [1], and reduced clinical and pathological disease severity in a mouse model of colitis [7]. Kuda et al. [5] also demonstrated that docosahexaenoic acid of 13-hydroxy linoleic acid (13-DHAHLA) suppressed LPS-induced cytokine secretion in the mouse macrophage cell line. In addition, Kolar et al. [9] showed that 13-LAHLA exhibited an anti-inflammatory effect by suppressing LPS stimulated cytokines, including IL-1β, IL-6, iNOS, and COX-2 in RAW 264.7 cells. In this study, we provided new findings that 9-POHSA and 9-OAHSA functioned as dexamethasone to suppressed LPS stimulated cytokines. To our knowledge, we are the first to report that 9-OAHSA and 9-POHSA also possessed an anti-inflammatory effect by suppressing LPS stimulated cytokines.

We found that the levels of 9-POHSA were higher than 9-OAHSA, and both demonstrated a significant positive correlation with POA levels and POA to PA ratio. We also found that SA levels indicated a positive correlation with 9-POHSA but not with 9-OAHSA levels. Although OA is an endogenous precursor of 9-OAHSA, OA level and OA to POA or SA ratios did not contribute to the biosynthesis of 9-OAHSA and 9-POHSA. Hence, these results indicated that the biosynthesis efficiency of 9-POHSA might be better than 9-OAHSA in healthy subjects. In addition, we observed that the circulating level of POA was the lowest among other fatty acids precursors. POA is an omega-7 monounsaturated fatty acid (MUFA) found in plants and marine sources, has been shown to favorably modulate lipid and glucose metabolism [48,49]. In animal models, dietary supplemented POA decreased the expression of pro-inflammatory cytokines TNF-α and IL-6 [50] and reduced atherosclerosis development [49]. More importantly, these results showed that POA level contributed to FAHFAs biosynthesis. These findings also imply the potential role of dietary POA in the prevention of CVD and diet-induced metabolic disorders.

**Figure 7:**
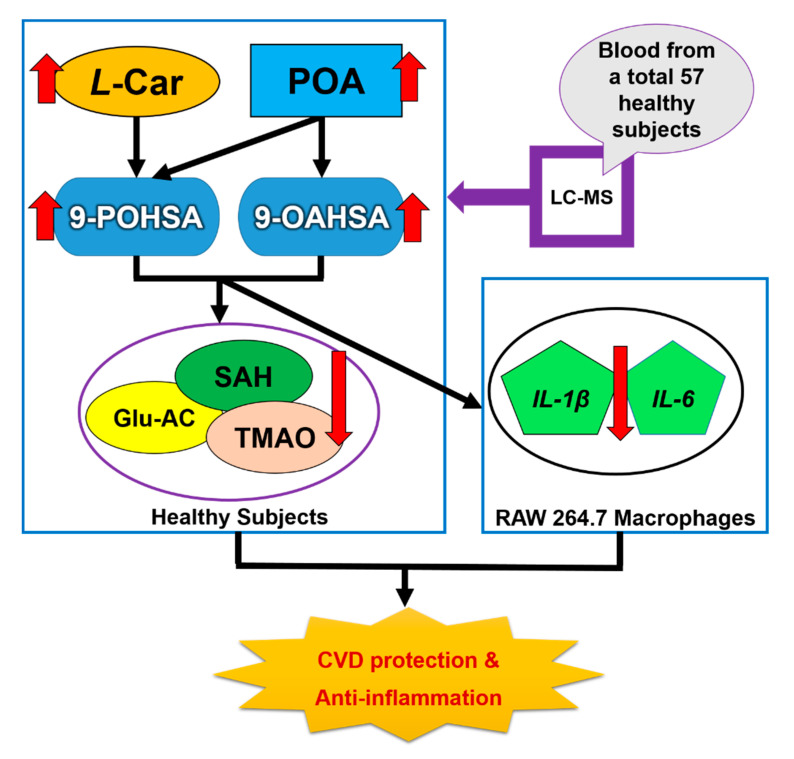
A schematic diagram depicting two major endogenous FAHFAs (9-POHSA and 9-OAHSA) in healthy human circulation, their CVD protection effects, and their anti-inflammatory effects on RAW 264.7 macrophages.

Taken together, 9-POHSA and 9-OAHSA possessed both CVD protection and anti-inflammatory effect (Figure 7). However, several limitations still need to be mentioned. First, the number of healthy subjects was small, the sample sizes could be increased and may affect the significance of this study. Second, since the correlation of 9-POHSA and 9-OAHSA with CV-related biomarkers have been uncovered in this study, there is a need to investigate their potency in CVD patient. Third, it remains to investigate other FAHFA types and their correlation with SAH, l-Car, TMAO, and other CV-related biomarkers.

## 5. Conclusions

In conclusion, we found that the levels of POA were lower than OA, on the contrary, the levels of 9-POHSA were higher than 9-OAHSA. These results indicated that the biosynthesis efficacy and the bioavailability of 9-POHSA might be better than 9-OAHSA in healthy subjects. In addition, the elevation of l-Car may promote the upregulation levels of 9-POHSA and further decrease the inflammation. Thereafter, the upregulation of the plasma 9-POHSA and 9-OAHSA levels may cause SAH and TMAO reduction to achieve the CVDs protection. 9-POHSA and 9-OAHSA also exhibited an anti-inflammatory effect by suppressing LPS stimulated cytokines, including *IL-1β* and *IL-6* gene expression in RAW 264.7. Therefore, we clearly presented evidence that FAHFAs may have potent protective effects against CVDs.

## Figures and Tables

**Table 1:** Baseline characteristics of 57 healthy subjects.

Variable	Subjects (Male *n* = 24, Female *n* = 33) Mean ± SD	Normal Range
Age (years old)	49.9 ± 12.4	n/a
Height (cm)	162.2 ± 8.5	n/a
Body weight (kg)	63.2 ± 13.2	n/a
Body mass index (BMI, kg/m^2^)	23.6 ± 3.3	n/a
Fasting blood glucose (mg/dL)	95.0 ± 25.7	<125
Triglyceride (TG, mg/dL)	122.4 ± 66.4	<200
Total cholesterol (TCH, mg/dL)	174.2 ± 31.9	<200
High-density lipoprotein cholesterol (HDL-C, mg/dL)	54.5 ± 13.7	>40
Low-density lipoprotein cholesterol (LDL-C, mg/dL)	101.0 ± 25.7	<130

n/a—not applicable.

**Table 2:** FAHFAs, CV-related biomarkers, and fatty acid levels determined in healthy subjects by using LC-MS.

FAHFAs, CV-Related Biomarkers, and Fatty Acids	Subjects (Male *n* = 24, Female *n* = 33) Mean ± SD
9-POHSA (nM)	1184.4 ± 526.1
9-OAHSA (nM)	374.0 ± 194.6
l-homocysteine (l-Hcy, µg/L)	1081.5 ± 479.8
*S*-adenosyl-*L*- homocysteine (SAH, µg/L)	8.7 ± 4.6
l-carnitine (l-Car, µg/L)	4576.0 ± 1724.5
Trimethylamine *N*-oxide (TMAO, µg/L)	273.5 ± 55.2
Palmitic acid (PA, µM)	322.1 ± 123.9
Stearic acid (SA, µM)	400.4 ± 190.6
Palmitoleic acid (POA, µM)	17.5 ± 11.0
Oleic acid (OA, µM)	226.1 ± 83.0

## Supplementary Materials

The following are available online at https://www.mdpi.com/2218-273X/10/12/1689/s1, Figure S1: The correlations of FAHFAs with their fatty acid precursors.
