# Supplementary material for: Determination of Major Endogenous FAHFAs in Healthy Human Circulation: The Correlations with Several Circulating Cardiovascular-Related Biomarkers and Anti-Inflammatory Effects on RAW 264.7 Cells
Source: Biomolecules. 2020 Dec 17;10(12):1689. doi: 10.3390/biom10121689 (PMC7766943; doi:10.3390/biom10121689)
Supplement: Supplementary file 1 [file biomolecules-10-01689-s001.pdf]

biomolecules

Figure Supplementary 1. The correlation of FAHFAs with their fatty acids precursors.

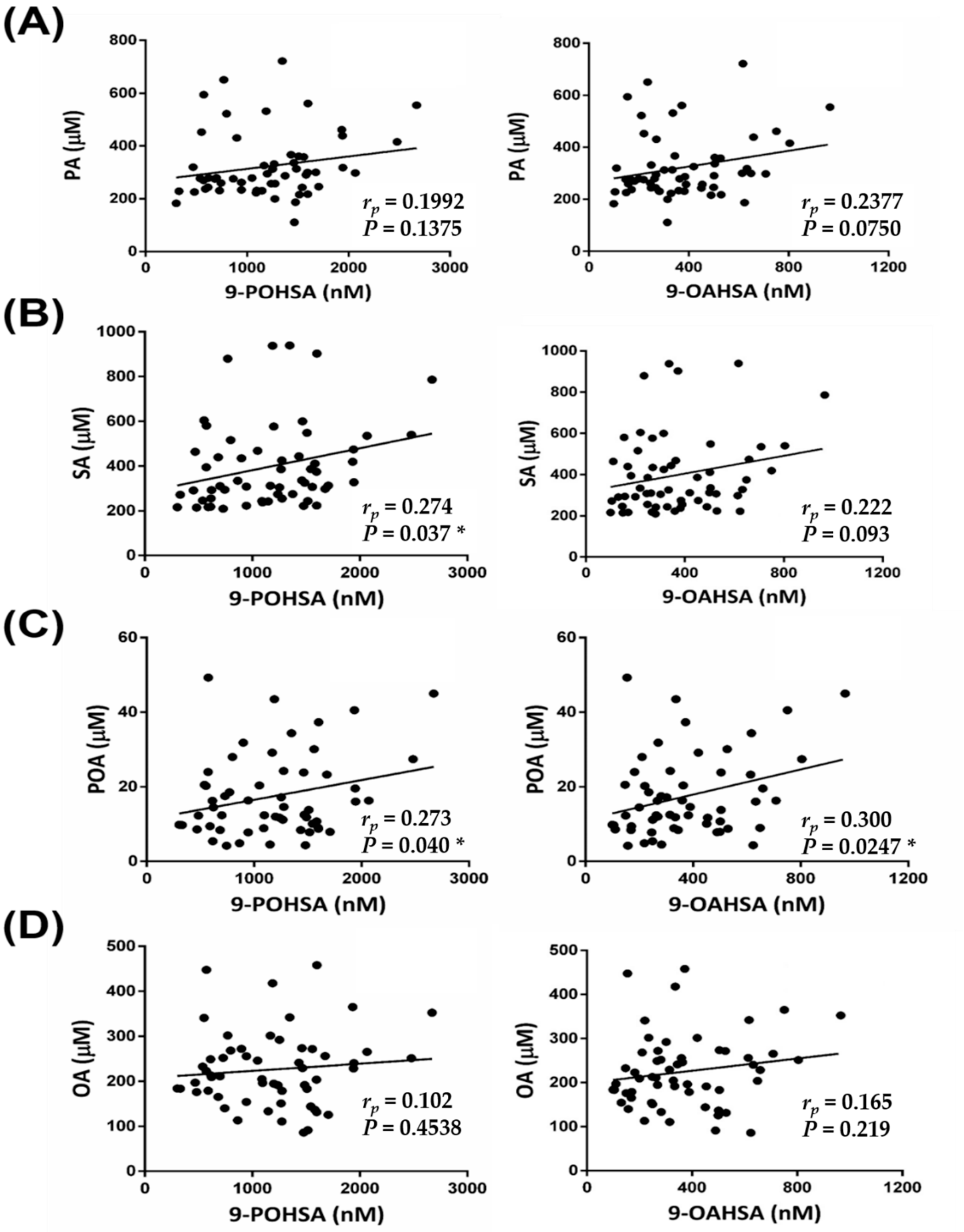

Figure S1. The correlation of FAHFAs with their fatty acids precursors. The correlation between PA, SA, POA, and OA with 9-POHSA and 9-OAHSA were shown in (**A**), (**B**), (**C**), and (**D**), respectively. POA level had a positive correlation with 9-POHSA and 9-OAHSA. r_p: Pearson correlation coefficient. Data are presented as mean ± SD (* $p < 0.05$).
